# Chromatin landscapes and genetic risk in systemic lupus

**DOI:** 10.1186/s13075-016-1169-9

**Published:** 2016-12-01

**Authors:** Joyce S. Hui-Yuen, Lisha Zhu, Lai Ping Wong, Kaiyu Jiang, Yanmin Chen, Tao Liu, James N. Jarvis

**Affiliations:** 1 Division of Pediatric Rheumatology, Steven and Alexandra Cohen Children’s Medical Center, 1991 Marcus Avenue, Suite M100, Lake Success, NY 11042 USA; 2 Department of Pediatrics, Hofstra-Northwell School of Medicine, Hempstead, NY 11549 USA; 3 Department of Biochemistry, University at Buffalo, Buffalo, NY 14203 USA; 4 Department of Pediatrics, University at Buffalo, Buffalo, NY 14203 USA; 5 Department of Biochemistry, and Genetics, Genomics, and Bioinformatics Program, University at Buffalo, Buffalo, NY 14203 USA; 6 Genetics, Genomics, and Bioinformatics Program, University at Buffalo, Buffalo, NY 14203 USA

**Keywords:** Systemic lupus erythematosus, Genetics, Enhancers, Neutrophils, Lymphocytes

## Abstract

**Background:**

Systemic lupus erythematosus (SLE) is a multi-system, complex disease in which the environment interacts with inherited genes to produce broad phenotypes with inter-individual variability. Of 46 single nucleotide polymorphisms (SNPs) shown to confer genetic risk for SLE in recent genome-wide association studies, 30 lie within noncoding regions of the human genome. We therefore sought to identify and describe the functional elements (aside from genes) located within these regions of interest.

**Methods:**

We used chromatin immunoprecipitation followed by sequencing to identify epigenetic marks associated with enhancer function in adult neutrophils to determine whether enhancer-associated histone marks were enriched within the linkage disequilibrium (LD) blocks encompassing the 46 SNPs of interest. We also interrogated available data in Roadmap Epigenomics for CD4^+^ T cells and CD19^+^ B cells to identify these same elements in lymphoid cells.

**Results:**

All three cell types demonstrated enrichment of enhancer-associated histone marks compared with genomic background within LD blocks encoded by SLE-associated SNPs. In addition, within the promoter regions of these LD blocks, all three cell types demonstrated enrichment for transcription factor binding sites above genomic background. In CD19^+^ B cells, all but one of the LD blocks of interest were also enriched for enhancer-associated histone marks.

**Conclusions:**

Much of the genetic risk for SLE lies within or near genomic regions of disease-relevant cells that are enriched for epigenetic marks associated with enhancer function. Elucidating the specific roles of these noncoding elements within these cell-type-specific genomes will be crucial to our understanding of SLE pathogenesis.

**Electronic supplementary material:**

The online version of this article (doi:10.1186/s13075-016-1169-9) contains supplementary material, which is available to authorized users.

## Background

Systemic lupus erythematosus (SLE) is a complex trait believed to be caused by gene–environment interactions that lead to a perturbed immunologic state in which autoantibodies, immune complex deposition, and complement activation contribute to systemic inflammation and target tissue damage. The genetics of systemic lupus has been studied extensively, in particular its association with complement deficiencies. Although rare, C1q deficiency is the strongest genetic risk factor for SLE [1, 2]. C1r and C1s deficiencies are commonly inherited together, and over 50% of these patients develop SLE [3]. Moreover, homozygous C2 and C4 deficiencies have been shown to predispose toward SLE [4–6].

Other than complement deficiencies, however, associations between SLE and functions of specific genes have been harder to clarify. This situation became even more confusing as data began to emerge from genome-wide association studies (GWAS) and genetic fine mapping studies [7–9], where the majority of risk-associated single nucleotide polymorphisms (SNPs) occurred in noncoding regions of the genome, often considerable distances (in genomic terms) from protein-coding genes and their promoters. Thus, while it is still common in the literature to identify disease-associated SNPs by their nearest gene, most genetic risk for SLE does not appear to be within “genes,” as conventionally understood, at all. In this respect, SLE resembles almost every other complex trait studied by GWAS [10]. Maurano et al. [10] have shown that most SNPs for most complex traits lie within genomic regions identified by projects like ENCODE, Roadmap Epigenomics, and Blueprint Epigenomics as regulatory regions, often regions active during fetal life. This observation has been confirmed from studies of specific diseases. Recently, for example, Jiang et al. [11] demonstrated that regions of genetic risk for juvenile idiopathic arthritis (JIA) identified by genetic fine mapping using Illumina Immunochip arrays are enriched for H3K4me1 and or H3K27ac histone marks, epigenetic signatures associated with enhancer function. There is thus a broadly emerging consensus in the fields of genetics and functional genomics that genetic risk for complex traits likely involves specific aspects of transcriptional regulation and coordination rather than aberrant function of protein-coding genes.

In the current study, we examined the “epigenetic landscape” around known SLE-associated SNPs in an effort to better understand the potential significance of disease-associated SNPs. We focused on three cell types known to contribute to SLE pathogenesis: CD19^+^ B cells, CD4^+^ T cells, and neutrophils [12–16]. We used ENCODE and Roadmap Epigenomics data as well as data generated in our own laboratory (for neutrophils) to identify functional elements within these regions.

## Methods

We queried the chromatin landscape around SNPs whose associations with SLE are well documented [17]. In addition, we queried recently reported SNPs found in a large Asian population [18]. CD19^+^ B-cell and CD4^+^ T-cell data were queried from ENCODE, while neutrophil RNA sequencing (RNAseq) and chromatin immunoprecipitation sequencing (ChIP-seq) data for H3K4me1/H3K27ac data were generated in our laboratory and have been reported recently [11]. Laboratory methods for ChIP-seq and RNAseq data are described briefly in the following.

### Healthy adults

Enhancers are both cell specific and cell-state specific [19]. Because neutrophils were not among the cells studied in either the ENCODE or Roadmap Epigenomics projects, we sought to create a genomic map for enhancer element locations using normal adult neutrophils. We obtained neutrophils from three healthy adults aged 25–40 using techniques we have described previously [11].

### Chromatin immunoprecipitation for histone marks H3K4me1 and H3K27ac and sequencing

Neutrophils were isolated as described previously [20]. The ChIP assay was carried out according to the protocol of the manufacturer (Cell Signaling Technologies Inc., Danvers, MA, USA) and has been described in our work published previously [11]. Briefly, adult neutrophils were incubated with newly prepared 1% formaldehyde in PBS at room temperature (RT). Crosslinking was quenched by adding 1× glycine. The crosslinked samples were centrifuged, the supernatant discarded, and the pellet washed with cold PBS followed by resuspension in 10 ml ice-cold Buffer A plus DTT, PMSF, and protease inhibitor cocktail. Cells were incubated on ice and then centrifuged at 4 °C to precipitate nucleus pellets, which were then resuspended in 10 ml ice-cold Buffer A plus DTT. The nucleus pellet was incubated with Micrococcal nuclease for 20 minutes at 37 °C with frequent mixing to digest DNA. Sonication of nuclear lysates was performed using a Sonic Dismembrator (FB-705; Fisher Scientific, Pittsburgh, PA, USA) on ice. After centrifugation of sonicated lysates, the supernatant was transferred into a fresh tube. Fifty microliters of the supernatant (chromatin preparation) was taken to analyze chromatin digestion and concentration. Fifteen micrograms of chromatin was added into 1× ChIP buffer plus protease inhibitor cocktail to a total volume of 500 μl. After removal of 2% of chromatin as the input sample, the antibodies were added to the ChIP buffer. The antibodies against respective histone modifications were rabbit polyclonal antibodies against histone H3 acetylated at lysine 27 (H3K27ac) and histone H3 monomethylated at lysine 4 (H3K4me1) from Cell Signaling Technologies. The negative control was normal IgG (Cell Signaling Technologies). After immunoprecipitation, the magnetic beads were added and incubated for another 2 hours at 4 °C. The magnetic beads are covalently coupled to a truncated form of recombinant protein G. They were then collected with a magnetic separator (Life Technologies, Grand Island, NY, USA). The beads were washed sequentially with low and high salt wash buffer, followed by incubation with elution buffer to elute protein/DNA complexes and reverse crosslinks of protein/DNA complexes to release DNA. The DNA fragments were purified by spin columns and dissolved in the elution buffer. The crosslinks of input sample were also reversed in elution buffer containing proteinase K before purification with spin columns. DNA sequencing was then conducted using the Illumina HiSeq 2500 at the next-generation sequencing center in University at Buffalo.

### ChIP-seq analysis of neutrophils

Analysis of the ChIP-seq data was carried out exactly as described previously [11]. MACS2 v2.1.10 [21] was applied for calling regions enriched with histone marks against the input sample, with the parameters “–nomodel --extsize 150 --broad –broad-cutoff 0.1”. Details of these analyses are further described by Jiang et al. [11].

### CD19^+^ B-cell and CD4^+^ T-cell analysis

In order to compare data from neutrophils with existing data from CD19^+^ B cells and CD4^+^ T cells, we queried data generated from the Roadmap Epigenomics Project [22]. Raw ChIP-seq data for CD19^+^ primary cells were downloaded from the GEO database [23] [GEO:GSM1027296, GEO:GSM1027287, GEO:GSM1027300, GEO:GSM1027304] for H3K4me1, H3K27ac, H3K4me3, and input control respectively. Raw ChIP-seq data for CD4^+^ T cells were downloaded [GEO:GSM1220567, GEO:GSM1220560, GEO:GSM1102798, GEO:GSM1102805] for H3K4me1, H3K27ac, H3K4me3, and input control respectively. The methods for mapping and region-calling are the same as those was used to analyze neutrophil data.

### ENCODE transcription factor binding site enrichment

ENCODE transcription factor binding site (TFBS) data were downloaded from UCSC Genome Browser ENCODE (http://hgdownload.cse.ucsc.edu/goldenPath/hg19/encodeDCC/wgEncodeRegTfbsClustered/). Only the TFBS information derived from blood cells was used for analysis. The whole genome was binned to 100 bp bins and intersected with H3K27ac, H3K4me1, or H3K4me3 peak regions, which were used as background. Fisher’s exact test was applied to test the significance of enrichment for TFBS for each transcription factor (TF) within H3K27ac, H3K4me1, or H3K4me3 peaks within linkage disequilibrium (LD) blocks compared with peak regions in the whole genome. The cutoff point for the false discovery rate (FDR) was set to 0.05.

## Results

### Association of regions of genetic risk with functional elements within neutrophil genomes

We searched the LD regions near (within 5 kb of) each GWAS index locus for association with histone marks from ChIP-seq. LD blocks were defined for 46 out of the 58 SNPs described in recent GWAS [17, 18] using information from the SNAP database (http://www.broadinstitute.org/mpg/snap) [24] by querying data from the 1000 Genomes Project pilot and the HapMap3 database. LD blocks were defined using *r* ^2^ < 0.9.

We further investigated H3K4me1, H3K4me3, and H3K27ac distal regions relative to transcription start sites. The distal regions typically correspond to *cis*-acting enhancers located far away from the gene(s) they regulate [25]. Regions containing at least one methylated (H3K4me1 or H3K4me3) and one acetylated histone mark (H3K27ac) were referred to as active enhancers, and those that contained only one methylated histone group or H3K27ac region were referred to as poised enhancers or H3K27ac-active enhancers, respectively. Of note, H3K4me3 appears to be cell-type specific, expressed in cells of the lymphoid lineage, and noted to be an important histone mark for enhancer activity [26].

**Fig. 1:**
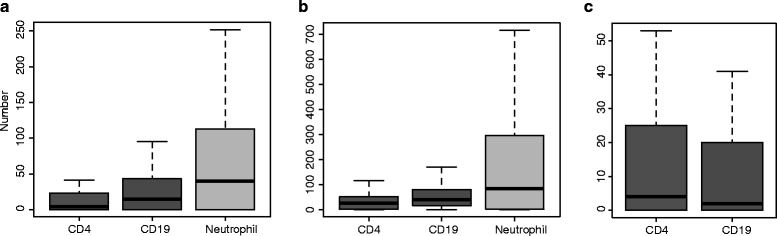
Number of H3K27ac, H3K4me1, and H3K4me3 peak regions (histone marks) in linkage disequilibrium blocks. **a** Box plot of the raw number of H3K27ac regions in CD4^+^ cells, CD19^+^ cells, and neutrophils. **b** Box plot of the raw number of H3K4me1 regions in CD4^+^ cells, CD19^+^ cells, and neutrophils. **c** Box plot of the raw number of H3K4me3 regions in CD4^+^ and CD19^+^ cells

We found functional elements within 5 kb of 36 of the 46 SNPs in adult neutrophils. Epigenetic evidence for active enhancers were found in 29 LD blocks, poised enhancers in six LD blocks, and H3K27ac-active enhancers in one LD block (Table 1). Using Fisher’s exact test, these regions were significantly enriched for enhancer activity above the genomic background (*p* < 0.05) (Fig. 1).

**Table 1:** Histone marks in the SNP linkage disequilibrium blocks in neutrophils

GWAS index SNP	Chr	Linkage disequilibrium blocks	Number of H3K27ac marks	Number of H3K4me1 marks	Enhancer marks (yes/no)
rs10028805	4	102736456–102762581	0	0	No
rs10036748	5	150457485–150461049	55	388	Yes
rs10488631	7	128585616–128711874	104	468	Yes
rs1059312	12	129277164–129288534	44	84	Yes
rs10774625	12	111884608–112007756	178	368	Yes
rs10807150	6	35154315–35278796	160	263	Yes
rs10936599	3	169477506–169528523	41	58	Yes
rs11644034	16	85967285–85980534	0	24	Yes
rs11889341	2	191943742–191970120	0	0	No
rs12022418	1	192521591–192535107	17	45	Yes
rs1270942	6	31704294–32175415	126	0	Yes
rs12802200	11	566936–567627	0	39	Yes
rs1610555	18	67523453–67544046	0	0	No
rs1801274	1	161470042–161479745	36	317	Yes
rs1885889	13	100084234–100104106	25	53	Yes
rs2009453	11	65399528–65405300	78	237	Yes
rs223881	16	57386566–57317134	0	0	No
rs2286672	17	4706123–4712617	0	3	Yes
rs2289583	15	75285114–75370012	227	386	Yes
rs2305772	19	52021247–52034940	0	0	No
rs2421184	5	158884119–158886939	0	0	No
rs2431697	5	159879978–159883217	0	0	No
rs2476601	1	114303808–114377568	113	297	Yes
rs2663052	10	50045456–50081232	0	0	No
rs2732549	11	35073852–35098193	26	106	Yes
rs2736340	8	11337587–11353000	0	0	No
rs2941509	17	37885383–38077485	308	693	Yes
rs3024505	1	206939904–206943968	0	66	Yes
rs34572943	16	31272353–31276811	79	150	Yes
rs3768792	2	213871709–213890232	0	0	No
rs3794060	11	71138710–71203790	62	125	Yes
rs4917014	7	50278187–50308811	66	65	Yes
rs4948496	10	63803472–63819903	67	83	Yes
rs61616683	22	39747050–39756650	0	22	Yes
rs6568431	6	106574794–106597639	15	15	Yes
rs6740462	2	65654364–65667272	26	35	Yes
rs6932056	6	138132123–138243700	175	286	Yes
rs740840	12	6327008–63600683	38	86	Yes
rs7444	22	21920817–21983260	153	420	Yes
rs7556469	1	198607998–198637582	379	487	Yes
rs7726414	5	133232663–13877357	252	1090	Yes
rs7941765	11	128499000–128500215	59	99	Yes
rs849142	7	28162674–28200097	17	222	Yes
rs9462027	6	34563164–34828553	167	717	Yes
rs9652601	16	11164567–11207894	63	105	Yes
rs9782955	1	235907825–236041129	322	511	Yes

Linkage disequilibrium blocks were obtained from the 1000 Genomes Project pilot 1 and/or HapMap3 database

*Chr* chromosome, *GWAS* genome-wide association studies, *SNP* single nucleotide polymorphism

### Enhancer elements within CD4^+^ T and CD19^+^ B cells

Using the same approaches as we used for neutrophils, we interrogated available H3K4me1, H3K4me3, and H3K27ac ChIP-seq data from the Roadmap Epigenomics project for CD4^+^ T and CD19^+^ B cells. We found considerable overlap for the locations of H3K4me1 and H3K27ac peaks between our neutrophil data and resting CD4^+^ T cells. Functional elements were found within 39 of the 46 SNPs of interest in CD4^+^ T cells. Epigenetic evidence for active enhancers was found in 30 LD blocks, and for poised enhancers in nine LD blocks; no LD blocks contained H3K27ac marks alone (Table 2).

**Table 2:** Histone marks in the SNP linkage disequilibrium blocks in CD4^+^ T cells

GWAS index SNP	Chr	Linkage disequilibrium blocks	Number of H3K27ac marks	Number of H3K4me1 marks	Number of H3K4me3 marks	Enhancer marks (yes/no)
rs10028805	4	102736456–102762581	0	0	0	No
rs10036748	5	150457485–150461049	18	35	22	Yes
rs10488631	7	128585616–128711874	11	30	14	Yes
rs1059312	12	129277164–129288534	2	19	0	Yes
rs10774625	12	111884608–112007756	2	15	0	Yes
rs10807150	6	35154315–35278796	89	116	53	Yes
rs10936599	3	169477506–169528523	30	22	36	Yes
rs11644034	16	85967285–85980534	13	31	6	Yes
rs11889341	2	191943742–191970120	0	9	0	Yes
rs12022418	1	192521591–192535107	0	0	0	No
rs1270942	6	31704294–32175415	29	69	228	Yes
rs12802200	11	566936–567627	0	0	0	No
rs1610555	18	67523453–67544046	0	1	0	Yes
rs1801274	1	161470042–161479745	0	0	6	Yes
rs1885889	13	100084234–100104106	20	38	10	Yes
rs2009453	11	65399528–65405300	5	17	3	Yes
rs223881	16	57386566–57317134	0	0	0	No
rs2286672	17	4706123–4712617	0	7	7	Yes
rs2289583	15	75285114–75370012	4	66	14	Yes
rs2305772	19	52021247–52034940	0	0	0	No
rs2421184	5	158884119–158886939	0	3	0	Yes
rs2431697	5	159879978–159883217	0	0	0	No
rs2476601	1	114303808–114377568	65	53	25	Yes
rs2663052	10	50045456–50081232	0	0	0	No
rs2732549	11	35073852–35098193	23	47	0	Yes
rs2736340	8	11337587–11353000	0	2	11	Yes
rs2941509	17	37885383–38077485	228	342	116	Yes
rs3024505	1	206939904–206943968	1	47	0	Yes
rs34572943	16	31272353–31276811	0	11	0	Yes
rs3768792	2	213871709–213890232	0	0	2	Yes
rs3794060	11	71138710–71203790	14	29	28	Yes
rs4917014	7	50278187–50308811	59	59	5	Yes
rs4948496	10	63803472–63819903	41	53	27	Yes
rs61616683	22	39747050–39756650	0	0	0	No
rs6568431	6	106574794–106597639	4	34	0	Yes
rs6740462	2	65654364–65667272	0	28	28	Yes
rs6932056	6	138132123–138243700	97	117	53	Yes
rs740840	12	6327008–63600683	1	25	0	Yes
rs7444	22	21920817–21983260	5	133	13	Yes
rs7556469	1	198607998–198637582	166	41	126	Yes
rs7726414	5	133232663–13877357	309	809	318	Yes
rs7941765	11	128499000–128500215	8	9	2	Yes
rs849142	7	28162674–28200097	1	25	2	Yes
rs9462027	6	34563164–34828553	68	110	66	Yes
rs9652601	16	11164567–11207894	9	78	0	Yes
rs9782955	1	235907825–236041129	22	33	24	Yes

Linkage disequilibrium blocks were obtained from the 1000 Genomes Project pilot 1 and/or HapMap3 database

*Chr* chromosome, *GWAS* genome wide association studies, *SNP* single nucleotide polymorphism

**Fig. 2:**
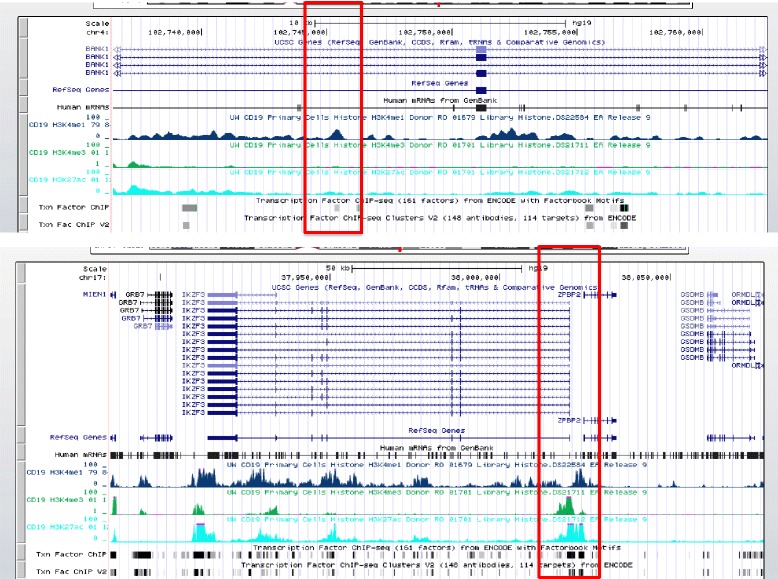
Representative screenshots of functional elements within CD19^+^ cell genomes generated from the University of California, Santa Cruz genome browser. *Red boxes* Potential active enhancers, showing signal peak regions in H3K4me1, H3K4me3, and/or H3K27ac. Upper panel LD block of SNP rs10028805. Genes within this block are noted. The LD block contains enhancer regions with multiple potential TFBSs (*gray* and/or *black bars* under the TF ChIP-seq track). Lower panel LD block of SNP rs2941509. Similar findings as in Fig. 1a are noted. ChIP data on the H3K4me1, H3K4me3, and H3K27ac enhancers are from the Roadmap Epigenomics project. *ChIP-seq* chromatin immunoprecipitation sequencing, *Chr* chromosome, *hg19* human genome 19, *ENCODE* Encyclopedia of Functional DNA Elements

In CD19^+^ B cells, we identified functional elements found in 42 of 46 SNPs of interest. Epigenetic evidence for active enhancers was present in 32 LD blocks, and for poised enhancers in 10 LD blocks; no LD blocks contained H3K27ac histone marks alone (Table 3). There are thus more SNPs of interest in SLE with functional elements within lupus-associated LD blocks in CD19^+^ cells than in either CD4^+^ cells or neutrophils (*p* < 0.05). Representative screenshots from the UCSC Genome Browser with two of the LD blocks of interest are shown in Fig. 2.

**Table 3:** Histone marks in the SNP linkage disequilibrium blocks in CD19^+^ B cells

GWAS index SNP	Chr	Linkage disequilibrium blocks	Number of H3K27ac marks	Number of H3K4me1 marks	Number of H3K4me3 marks	Enhancer marks (yes/no)
rs10028805	4	102736456–102762581	44	61	13	Yes
rs10036748	5	150457485–150461049	15	25	14	Yes
rs10488631	7	128585616–128711874	43	62	11	Yes
rs1059312	12	129277164–129288534	33	66	1	Yes
rs10774625	12	111884608–112007756	0	34	0	Yes
rs10807150	6	35154315–35278796	71	110	33	Yes
rs10936599	3	169477506–169528523	32	18	33	Yes
rs11644034	16	85967285–85980534	49	106	23	Yes
rs11889341	2	191943742–191970120	0	0	0	No
rs12022418	1	192521591–192535107	0	0	0	No
rs1270942	6	31704294–32175415	57	58	123	Yes
rs12802200	11	566936–567627	0	0	0	No
rs1610555	18	67523453–67544046	0	0	0	No
rs1801274	1	161470042–161479745	0	8	0	Yes
rs1885889	13	100084234–100104106	17	43	3	Yes
rs2009453	11	65399528–65405300	0	12	0	Yes
rs223881	16	57386566–57317134	0	30	0	Yes
rs2286672	17	4706123–4712617	1	7	7	Yes
rs2289583	15	75285114–75370012	23	122	8	Yes
rs2305772	19	52021247–52034940	2	35	11	Yes
rs2421184	5	158884119–158886939	0	18	0	Yes
rs2431697	5	159879978–159883217	0	2	0	Yes
rs2476601	1	114303808–114377568	18	32	13	Yes
rs2663052	10	50045456–50081232	0	1	0	Yes
rs2732549	11	35073852–35098193	6	47	0	Yes
rs2736340	8	11337587–11353000	40	71	29	Yes
rs2941509	17	37885383–38077485	235	468	84	Yes
rs3024505	1	206939904–206943968	0	48	0	Yes
rs34572943	16	31272353–31276811	0	36	0	Yes
rs3768792	2	213871709–213890232	0	10	0	Yes
rs3794060	11	71138710–71203790	10	28	22	Yes
rs4917014	7	50278187–50308811	17	57	0	Yes
rs4948496	10	63803472–63819903	80	101	17	Yes
rs61616683	22	39747050–39756650	5	17	0	Yes
rs6568431	6	106574794–106597639	4	14	0	Yes
rs6740462	2	65654364–65667272	1	20	27	Yes
rs6932056	6	138132123–138243700	57	115	20	Yes
rs740840	12	6327008–63600683	0	26	0	Yes
rs7444	22	21920817–21983260	14	88	10	Yes
rs7556469	1	198607998–198637582	47	81	26	Yes
rs7726414	5	133232663–13877357	540	1176	284	Yes
rs7941765	11	128499000–128500215	19	45	0	Yes
rs849142	7	28162674–28200097	45	120	0	Yes
rs9462027	6	34563164–34828553	95	171	41	Yes
rs9652601	16	11164567–11207894	31	82	0	Yes
rs9782955	1	235907825–236041129	38	71	15	Yes

Linkage disequilibrium blocks were obtained from the 1000 Genomes Project pilot 1 and/or HapMap3 database

*Chr* chromosome, *GWAS* genome-wide association studies, *SNP* single nucleotide polymorphism

While it is difficult at this time to determine exact differences in the acetylation and methylation of patients with SLE compared with healthy controls due to the scarcity of available data, we did perform an analysis of differentially methylated regions as identified by Absher et al. [27] in adult SLE patients. This analysis revealed only one gene (PBX2, on chromosome 6) lying within the same LD block as rs1270942 to be aberrantly methylated in T cells, B cells, and/or monocytes in SLE patients. In addition, analysis of differentially methylated regions identified by Coit et al. [28] in the neutrophils of adult SLE patients compared with healthy adult data from our laboratory and available in Roadmap Epigenomics revealed that none of these regions were located within the LD blocks containing the SLE-associated SNPs. Less than 40% of these regions contained enhancer marks in healthy adult neutrophils. Fewer than 1/3 of these regions contained enhancer marks in healthy adult CD4^+^ and CD19^+^ cells.

### Transcription factor binding sites at enhancer regions in LD blocks in CD4^+^ cells, CD19^+^ cells, and neutrophils

**Fig. 3:**
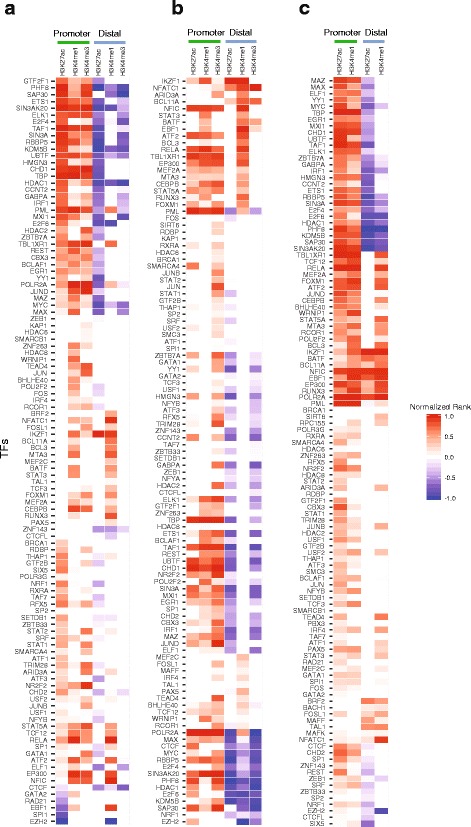
Enrichment of TFBSs in enhancer regions. **a** Heatmap of TFBSs in H3K27ac, H3K4me1, and H3K4me3 peak regions in promoter and distal regions in CD19^+^ cells. **b** Heatmap of TFBS in H3K27ac, H3K4me1, and H3K4me3 peak regions in promoter and distal regions in CD4^+^ cells. **c** Heatmap of TFBSs in H3K27ac and H3K4me1 peak regions in promoter and distal regions in neutrophils. Normalized rank: > 0 enrichment (*red*); <0 depletion (*blue*). *TF* transcription factor (Color figure online)

We next sought to further test the likely functional significance of H3K4me1, H3K4me3, and H3K27ac enrichment within the SLE-associated LD blocks. We therefore analyzed the TF ChIP-seq data from blood cells obtained from the UCSC Genome Browser ENCODE data portal [29] to determine whether there was significant enrichment for TF binding within the enhancer regions located within SLE-associated LD blocks compared with other regions (Fig. 3). We investigated both promoter and nonpromoter regions within the regions where histone marks (H3K4me1, H3K4me3, and H3K27ac) are associated with enhancer activities. As expected, promoter regions within these LD blocks (defined as (−5 K, 1 K) of transcription start sites) are highly enriched for TF binding sites. Furthermore, we identified more enrichment for TF binding sites in H3K4me3 peak regions than in either H3K27ac or H3K4me1 peak regions in promoter sites in CD19^+^ and CD4^+^ T cells (*p* < 0.05). Overall, the LD blocks containing lupus-associated SNPs appeared to be in active, dynamic regions of leukocyte genomes as determined by the abundance of transcription factor binding motifs within these regions

Of note, 23 out of 46 SNP regions have shared histone modifications in all three cell types investigated. There are 102 genes located within the 46 SNP LD blocks, which are involved in 26 Panther pathways, including T-cell and B-cell activation, and Jak/STAT signaling pathways (Additional file 1: Table S1). Moreover, Farh et al. [30] demonstrated that many causal variants which map to immune-cell enhancers may gain histone acetylation and transcribe enhancer-associated RNA upon immune stimulation. This could indicate that the majority of SNPs are functional SNPs. However, expression quantitative loci analysis (eQTL) revealed that only one SNP (rs2736340) was associated with a target gene (FAM167A). Similarly, when the SNPs of interest were compared with genes identified by Bennett et al [31]. whose expression was upregulated in SLE patients, only one gene was found to lie in the same LD block as one of the identified SNPs: a phorbolin-1 like gene from the interferon family lies in the same LD block as SNP rs61616683.

## Discussion

Multiple GWAS in human disease have yielded surprising data demonstrating that a significant majority of disease-associated polymorphisms are located within noncoding regions of the genome [8, 9], i.e., those regions of the genome where transcription is coordinated on a genome-wide basis [32]. In fact, only 1–2% of the human genome is believed to span protein-coding genes [33, 34] and the remaining DNA is believed to incorporate an abundance of regulatory elements that contribute to maintenance of a cell’s identity and/or regulate specific cell functions.

Results of recent GWAS in SLE also illustrate this point. Of 46 SNPs identified by Bentham et al. and Sun et al. [17, 18] as conferring risk for SLE, only 16 lie within coding regions. In this study, we demonstrate that these SLE-associated SNPs lie within LD blocks containing histone marks commonly associated with enhancer function. We identified these marks in three cell types known to contribute to SLE pathogenesis and/or disease manifestations: CD4^+^ T cells, CD19^+^ B cells, and neutrophils.

Enhancers are *cis*-acting, active regions of DNA that promote gene transcription. Enhancers act by binding transcription factors and other transcriptional regulators that then alter the three-dimensional conformation of chromatin and facilitate the interaction between gene promoters and protein–DNA complexes. Enhancers may lie considerable distances from the promoters they regulate and may not regulate the genes closest to them [35]. Enhancers may also be cell-type specific and tissue specific, and may regulate more than one gene [19, 36]. For example, Martin et al. [35] recently used HiC chromosome capture approaches to identify long-range interactions between autoimmune disease risk loci and target genes. They demonstrated that SNPs lying large distances apart (in genomic terms) might either interact with the nearest gene or bypass multiple genes lying nearer to them to interact with those situated more distally.

The finding that the genetic risk for SLE lies largely within functional, noncoding regions of the human genome that contain regulatory elements in neutrophils, CD4^+^ T cells, and CD19^+^ B cells invites a new perspective in disease pathogenesis. All three cell types have been implicated in the pathogenesis of SLE [12–16]. Preliminary studies in adult SLE used RNAseq and found differentially expressed genes comprising different cellular functions from distinct leukocyte populations (in particular, from B cells and monocytes) [37]. Thus, cell-type-specific differences in gene expression may contribute to the pathogenesis of SLE. Similar findings of risk in the noncoding genome have been observed in the neutrophils and CD4^+^ T cells of JIA patients [11].

Our results demonstrate that, particularly in lymphocytes, there is copious transcription factor binding in H3K4me1/H3K4me3/H327ac-marked regulatory regions encompassed by the LD blocks containing SLE-associated SNPs, providing further evidence that these are important regulatory regions. Shi et al. [38] have shown that both promoters and enhancers exhibit significant changes in monocytes from SLE patients when compared with healthy controls. In particular, differentially methylated regions in SLE were significantly enriched in potential interferon-related TFBS. Furthermore, the importance of histone modifications (e.g., epigenetic marks) in regulating transcription is demonstrated in the recent work of Zhang et al. These authors identified distinct patterns of H3K4me3 methylation associated with aberrations in gene expression in monocytes from patients with SLE [39]. Their results demonstrated that genes overexpressed in SLE tended to respond to H3K4me3 changes downstream of transcription start sites.

Our findings, as well the expanded understanding of gene regulation that has emerged in the past 10 years, suggest a new paradigm of SLE pathogenesis that includes complex interactions between the innate and adaptive immune systems that may emerge because of disordered transcriptional regulation in both lymphoid and myeloid cells. The field of functional genomics is demonstrating that transcription is a complex process that must be regulated and coordinated on a genome-wide basis to maintain normal cellular function [32]. For example, transcription factors do not simply bind to DNA independently from other proteins, but rather interact with one another in layers of complexity. This newer finding suggests that transcriptional and regulatory networks are created for complex biological processes, and that even small perturbations of this system (e.g., from genetic variance or environmentally-induced epigenetic alterations) could accumulate over time. These cumulative small perturbations result in significant disorders in the regulation and coordination of transcription, ultimately leading to the development of disease. These newer data suggest that complex disorders may be due less to “bad genes” than to faulty gene regulation [40, 41]. Perhaps SLE, and many of the other conditions we refer to as “autoimmune diseases,” may be better understood as a disease of disordered transcription.

It is important to keep in mind the limitations of our study. First, both Bentham et al. and Sun et al. [17, 18] focused on regions of immunologic interest when performing GWAS, in that both groups used the Illumina Immunochip. The GWAS thus identified only selected genomic regions of specific immunologic interest that confer risk for SLE. It will be important to investigate whether additional risk loci would be revealed with a broadening of the query to genes that regulate chromatin access, or genes that regulate specific epigenetic processes (e.g., DNA methyl transferases, histone deacetylases, etc.).

In addition, as already mentioned briefly, enhancers appear to be cell-type specific and tissue specific. The enhancer marks used in this study for neutrophils and lymphocytes as mapped by the Roadmap Epigenomics project were detected in adult blood cells. There is the possibility that slightly different results could be obtained from cells in pediatric SLE patients. Thus, our results may not be generalizable or extrapolated, for example, to a pediatric population without further investigation. Indeed, we already know that the epigenomes of pathologically relevant cells may differ from epigenetic marks annotated in Roadmap data [42]. Coit et al. [40], for example, have shown that the methylome in CD4^+^ T cells of patients with SLE shows distinct differences from what is observed in healthy controls.

Moreover, the SLE patients included in the GWAS studies were generated from heterogeneous populations and most likely included patients with varying clinical manifestations. It is well known that SLE can affect different organ systems in the body with varying severity, and that treatment of these manifestations varies from milder immunosuppression with hydroxychloroquine alone to more aggressive immunosuppression with anti-neoplastic agents for disease control. Recently, Haddon et al. [43] demonstrated that pediatric SLE patients with kidney involvement possessed a different autoantibody profile than those without kidney involvement. Other groups then showed differences in expression levels of RNA and microRNAs in lupus nephritis biopsies [44, 45], suggesting that different clinical phenotypes may have individualized gene expression signatures.

Our results also have implications for the optimization of therapy in patients with SLE. Jiang et al. [20] demonstrated that response to treatment in JIA suggests that children on treatment for JIA can experience a long period of asymptomatic disease remission, but continue to have immune cell dysregulation. Our current treatments for SLE do not “normalize” immune cell function, as evidenced by continued periods of disease remission and flare. A better understanding of how epigenetic signatures drive gene expression signatures in SLE patients will allow us to better determine which patients with which SLE phenotypes will respond best to different treatments given their epigenetic profiles.

## Conclusion

In this study, we have shown that disease-associated SNPs in SLE lie within LD blocks rich in functional elements regulating and coordinating gene transcription. These findings provide new insight into possible links between genetic and epigenetic risk factors for SLE.

## Additional file

Additional file 1: Table S1. is presenting genes (a) and functional pathways (b), as identified through Panther, associated with SNPs in systemic lupus. (DOCX 16 kb)

## Abbreviations

ChIP: Chromatin immunoprecipitation
ChIP-seq: Chromatin immunoprecipitation sequencing
FDR: False discovery rate
GWAS: Genome-wide association studies
JIA: Juvenile idiopathic arthritis
LD: Linkage disequilibrium
RNAseq: Ribnonucleotide sequencing
RT: Room temperature
SLE: Systemic lupus erythematosus
SNP: Single nucleotide polymorphism
TF: Transcription factor
TFBS: Transcription factor binding sites
