# Supplementary material for: Chromatin landscapes and genetic risk in systemic lupus
Source: Arthritis Res Ther. 2016 Dec 1;18:281. doi: 10.1186/s13075-016-1169-9 (PMC5134118; doi:10.1186/s13075-016-1169-9)
Supplement: Additional file 1: Table S1. — is presenting genes (a) and functional pathways (b), as identified through Panther, associated with SNPs in systemic lupus. (DOCX 16 kb) [file 13075_2016_1169_MOESM1_ESM.docx]

Additional file 1: Table S1. Genes (a) and functional pathways (b), as identified through Panther, associated with single nucleotide polymorphisms in systemic lupus.

1. Genes associated with SNPs in systemic lupus

SNP Possible associated gene SNP Possible associated gene

| rs1059312 | SLC15A4 | rs1270942 | VWA7 |
| --- | --- | --- | --- |
| rs10807150 | SCUBE3 | rs1270942 | VARS |
| rs10807150 | ZNF76 | rs1270942 | LSM2 |
| rs10807150 | DEF6 | rs1270942 | HSPA1L |
| rs10936599 | ACTRT3 | rs1270942 | HSPA1A |
| rs10936599 | MYNN | rs1270942 | HSPA1B |
| rs10936599 | LRRC34 | rs1270942 | C6orf48 |
| rs1610555 | CD226 | rs1270942 | NEU1 |
| rs1801274 | FCGR2A | rs1270942 | SLC44A4 |
| rs2009453 | PCNXL3 | rs1270942 | EHMT2 |
| rs223881 | CCL22 | rs1270942 | C2 |
| rs223881 | CX3CL1 | rs1270942 | ZBTB12 |
| rs2286672 | PLD2 | rs1270942 | CFB |
| rs2305772 | SIGLEC6 | rs1270942 | NELFE |
| rs2941509 | ERBB2 | rs1270942 | SKIV2L |
| rs2941509 | MIEN1 | rs1270942 | DXO |
| rs2941509 | GRB7 | rs1270942 | STK19 |
| rs2941509 | IKZF3 | rs1270942 | C4A |
| rs2941509 | ZPBP2 | rs1270942 | AL645922.1 |
| rs2941509 | GSDMB | rs1270942 | C4B |
| rs2941509 | ORMDL3 | rs1270942 | CYP21A2 |
| rs3768792 | IKZF2 | rs1270942 | TNXB |
| rs3794060 | DHCR7 | rs1270942 | ATF6B |
| rs3794060 | NADSYN1 | rs1270942 | FKBPL |
| rs61616683 | SYNGR1 | rs1270942 | PRRT1 |
| rs7726414 | C5orf15 | rs1270942 | PPT2 |
| rs7726414 | VDAC1 | rs1270942 | PPT2-EGFL8 |
| rs7726414 | TCF7 | rs1270942 | EGFL8 |
| rs7726414 | SKP1 | rs1270942 | AGPAT1 |
| rs7726414 | CTD-2410N18.5 | rs1270942 | RNF5 |
| rs7726414 | PPP2CA | rs1270942 | AGER |
| rs7726414 | CDKL3 | rs1270942 | PBX2 |
| rs7726414 | UBE2B | rs1270942 | GPSM3 |
| rs7726414 | CDKN2AIPNL | rs1270942 | NOTCH4 |
| rs7726414 | PHF15 | rs11889341 | STAT4 |
| rs2476601 | RSBN1 | rs10774625 | SH2B3 |
| rs2476601 | PTPN22 | rs10774625 | ATXN2 |
| rs7556469 | PTPRC | rs10488631 | IRF5 |
| rs9782955 | LYST | rs10488631 | TNPO3 |
| rs9652601 | CLEC16A | rs10036748 | TNIP1 |
| rs9462027 | C6orf106 | rs10028805 | BANK1 |
| rs9462027 | SNRPC | rs7726414 | CTD-2410N18.4 |
| rs9462027 | UHRF1BP1 |  |  |
| rs849142 | JAZF1 |  |  |
| rs7444 | UBE2L3 |  |  |
| rs7444 | YDJC |  |  |
| rs740840 | CD9 |  |  |
| rs6932056 | TNFAIP3 |  |  |
| rs6740462 | SPRED2 |  |  |
| rs4948496 | ARID5B |  |  |
| rs34572943 | ITGAM |  |  |
| rs3024505 | IL10 |  |  |
| rs2736340 | BLK |  |  |
| rs2663052 | WDFY4 |  |  |
| rs2289583 | SCAMP5 |  |  |
| rs2289583 | PPCDC |  |  |
| rs1270942 | CLIC1 |  |  |
| rs1270942 | MSH5 |  |  |
| rs1270942 | MSH5-SAPCD1 |  |  |
| rs1270942 | SAPCD1 |  |  |

1. Functional pathways associated with SNPs in systemic lupus

Functional Pathway Number of genes identified within SNPs

| JAK/STAT signaling pathway | 2 |
| --- | --- |
| Apoptosis signaling pathway | 4 |
| Angiogenesis | 4 |
| Interleukin signaling pathway | 2 |
| Alzheimer disease-presenilin pathway | 2 |
| Opioid prodynorphin pathway | 1 |
| Integrin signalling pathway | 1 |
| Inflammation mediated by chemokine and cytokine signaling pathway | 3 |
| Ubiquitin proteasome pathway | 2 |
| Coenzyme A biosynthesis | 1 |
| p53 pathway feedback loops 2 | 1 |
| p53 pathway by glucose deprivation | 1 |
| EGF receptor signaling pathway | 3 |
| Parkinson disease | 6 |
| Gonadotropin-releasing hormone receptor pathway | 2 |
| Vasopressin synthesis | 1 |
| PDGF signaling pathway | 1 |
| Ras Pathway | 1 |
| Notch signaling pathway | 1 |
| Cadherin signaling pathway | 1 |
| B cell activation | 2 |
| p53 pathway | 1 |
| Wnt signaling pathway | 2 |
| Toll receptor signaling pathway | 1 |
| T cell activation | 1 |
| FGF signaling pathway | 1 |
